# A metamaterial-free fluid-flow cloak

**DOI:** 10.1093/nsr/nwab205

**Published:** 2021-11-17

**Authors:** Fuyang Tay, Youming Zhang, Hongyi Xu, Honghui Goh, Yu Luo, Baile Zhang

**Affiliations:** Division of Physics and Applied Physics, School of Physical and Mathematical Sciences, Nanyang Technological University, Singapore 637371, Singapore; Division of Physics and Applied Physics, School of Physical and Mathematical Sciences, Nanyang Technological University, Singapore 637371, Singapore; Division of Physics and Applied Physics, School of Physical and Mathematical Sciences, Nanyang Technological University, Singapore 637371, Singapore; Division of Physics and Applied Physics, School of Physical and Mathematical Sciences, Nanyang Technological University, Singapore 637371, Singapore; School of Electrical and Electronic Engineering, Nanyang Technological University, Singapore 639798, Singapore; Division of Physics and Applied Physics, School of Physical and Mathematical Sciences, Nanyang Technological University, Singapore 637371, Singapore; Centre for Disruptive Photonic Technologies, Nanyang Technological University, Singapore 637371, Singapore

**Keywords:** invisibility cloaks, metamaterials, fluid-flow control

## Abstract

The model of ideal fluid flow around a cylindrical obstacle exhibits a long-established physical picture, where originally straight streamlines are deflected over the whole space by the obstacle. Inspired by transformation optics and metamaterials, recent theories have proposed the concept of fluid cloaking, which is able to recover the straight streamlines, as if the obstacle did not exist. However, such a cloak, similar to all previous transformation-optics-based devices, relies on complex metamaterials with inhomogeneous parameters and is difficult to implement. Here we deploy the theory of scattering cancellation and report on the experimental realization of a fluid-flow cloak without metamaterials. This cloak is realized by engineering the geometry of the fluid channel, which effectively cancels the dipole-like scattering of the obstacle. The cloaking effect is demonstrated through the direct observation of recovered straight streamlines in the fluid flow. Our work sheds new light on conventional fluid control and may find application in microfluidic devices.

## Teaser

By judiciously engineering the geometry of the fluid channel, an `invisibility' cloak able to make an object undetectable from fluid flow is realized experimentally.

## INTRODUCTION

Ideal fluid flow around a cylinder is a fundamental problem discussed in many textbooks on fluid mechanics [1]. Being inviscid and incompressible, an ideal fluid satisfies the mass continuity equation, which can be simplified into Laplace's equation at steady states. When encountering a circular cylinder, the ideal fluid no longer follows straight streamlines, but flows around the cylinder with deflected streamlines that are described by a conformal mapping [1]. This model provides a physical picture as a way to understand general fluid flow in fluid mechanics when a complex-shaped obstacle or fluid viscosity is involved.

Recently, with the inspiring development of transformation optics and metamaterials, substantial interest has arisen in constructing invisibility cloaking devices that are able to hide an object from external physical fields. These kinds of transformation-optics cloaking devices were firstly proposed and realized for electromagnetic waves [2–13], and then extended to acoustic waves, heat flow and other fields or waveforms [14–22]. In 2011, this concept was further extended to fluid flow [23]. However, similar to all previous transformation-optics devices, the design of this fluid-flow cloak exhibits spatially variant material parameters that require complex metamaterials, and thus is difficult to realize [24–29]. For example, the recent implementation of such a transformation-based fluid-flow cloak relied on 10 layers of metamaterial microstructures, as well as a fluid background filled with microcylinders to avoid impedance mismatch [25].

Scattering cancellation is another powerful approach for cloak design. Originally proposed for plasmonic particles in quasistatic electric fields [30], it has recently been extended to magnetic fields [31–33] and heat conduction [34,35]. However, the possibility of its application in fluid control has never been discussed.

Here we apply the approach of scattering cancellation to fluid control, and construct a fluid-flow cloak that is capable of hiding a cylindrical obstacle without disturbing the straight external streamlines (see the movie in Supplementary Data demonstrating the effectiveness of such a fluid cloak). In particular, the use of scattering cancellation in fluid flow has an unprecedented feature, i.e. being ‘metamaterial-free’—our fluid cloak is realized by changing the local geometry of the fluid channel, rather than employing any complex metamaterial design. By injecting dye particles into the fluid flow, we have directly observed the successful recovery of straight streamlines passing through the obstacle, as if the obstacle did not exist.

## DESIGNING A FLUID-FLOW CLOAK

We shall firstly point out that the ideal fluid with zero viscosity does, in fact, not exist in nature (it is ‘dry water’, as stated by John von Neumann [36]). The flow of a real fluid with finite viscosity is governed by the Navier-Stokes equation, which has nearly no analytical solution due to its non-linear viscosity term. Nevertheless, a viscous fluid flow in a narrow gap between two parallel plates, known as Hele-Shaw flow, can be described by a scalar potential function, exhibiting similar features of two-dimensional (2D) ideal fluid flow [1]. Moreover, Hele-Shaw flow plays a significant role in microfluidic devices and plastic-forming manufacturing operations, where a realistic cloak may find useful application.

**Figure 1.**
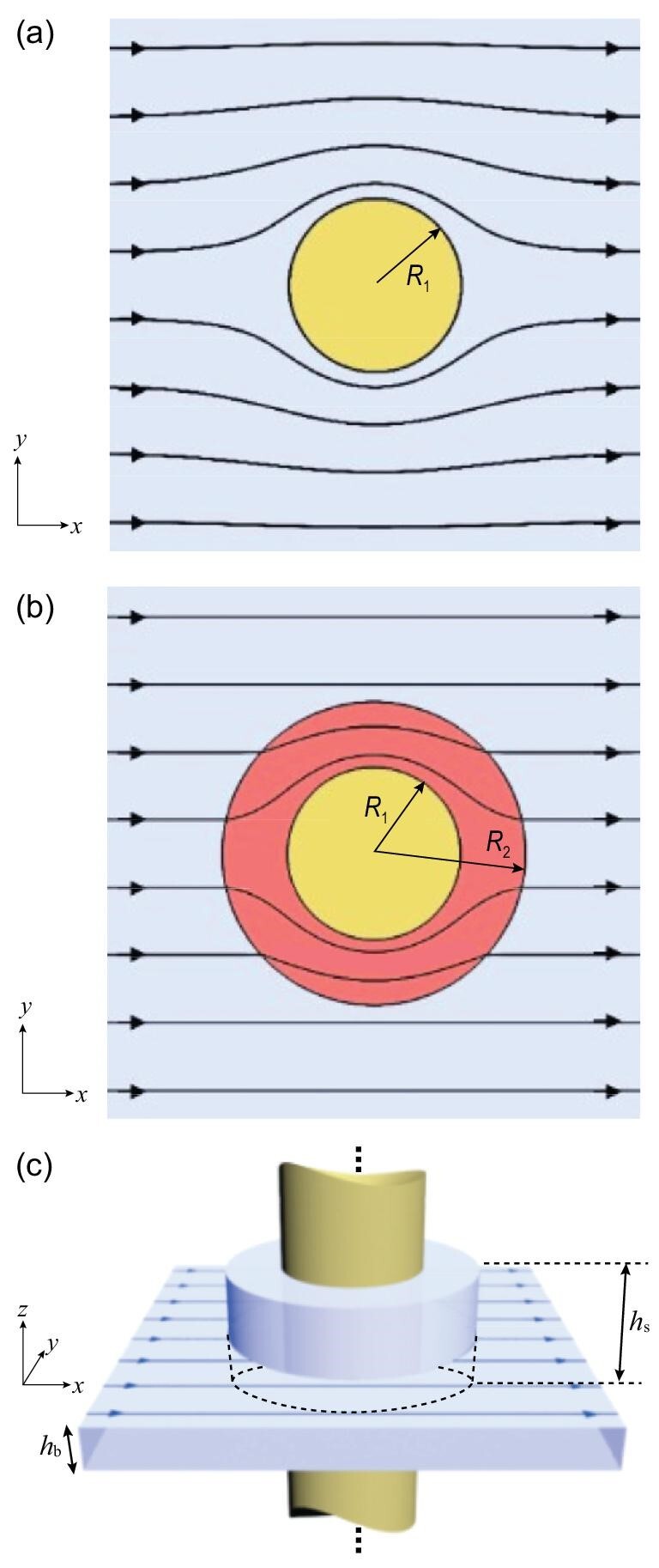
Design of a fluid cloak. (a) Streamlines for 2D ideal fluid flow around a circular cylinder with a radius ${R_1}$. (b) A hypothetical fluid cloak that can guide fluid flow around the cylindrical obstacle without disturbing the external straight streamlines. The cloak has an outer radius ${R_2}$ and inner radius ${R_1}$. (c) Conceptual illustration of a fluid cloak for the flow in a narrow fluid channel with a height of ${h_b}$. The fluid cloak is realized by increasing the height of the fluid channel to ${h_s}$.

Let us start with Fig. 1a, which depicts the ideal fluid flow around a cylinder with a radius ${R_1}$ in a 2D geometry [1]. By denoting the stream function as $\psi $ and the velocity potential as $\phi $ (here $\nabla \phi $ gives the flow velocity $\bar{v}$; $\psi $ and $\phi $ satisfy Cauchy-Riemann conditions), we can describe the flow with a complex potential $w\ ( z ) = \ \phi ( {x,\ y} ) + i\psi ( {x,\ y} )$ in the complex plane $z\ = \ x + iy$ [1]. Note that the velocity equipotential lines (i.e. constant $\phi $) and the streamlines (i.e. constant $\psi $) are orthogonal to each other. Therefore, either the real part or the imaginary part of the complex potential is sufficient to describe the ideal flow. Without the cylinder, the original uniform flow with straight streamlines exhibits the complex potential $w\ = \ Uz$, where *U* is the speed of the stream that flows uniformly in the $\hat{x}$ direction. The presence of the cylinder deflects the flow according to the conformal mapping of $z \to z + R_1^2/z$ [1]. Thus, the complex potential$\ w$ is mapped to $w^{\prime} = \ U( {z + R_1^2/z} )$ in the region outside of the cylinder (i.e. $| z | > {R_1}$). Note that the newly produced term $UR_1^2/z$ corresponds to the complex potential of a ‘dipole’-like doublet (i.e. a point source and a point sink placed extremely close to each other, similar to a dipole of positive charge and negative charge in electromagnetics), which has the dipole strength vector $- 2\pi UR_1^2\hat{x}$. Therefore, in the language of electromagnetics, the cylinder induces a ‘dipole field’ of fluid flux, disturbing the flow over the entire space.

This similarity to electromagnetics implies that it is possible to construct a fluid cloak by applying scattering cancellation. Indeed, we can consider a cloak as shown in Fig. 1b, which consists of a shell with an outer radius ${R_2}$ and an inner radius ${R_1}$ that encloses the cylindrical obstacle completely. This cloak can guide the fluid flux smoothly around the obstacle, leaving the external fluid flux undisturbed. In previous magnetic/thermal cloaks [31,34,35] the 2D calculation from Laplace's equation requires their magnetic permeability/thermal conductivity to take the relative value of $(R_2^2 + R_1^2)/( {R_2^2 - R_1^2} )$. By the same token, we can simply write down the mathematical condition of the fluid cloak as

(1) \begin{equation*} {\rho _2} = \frac{{R_2^2 + R_1^2}}{{R_2^2 - R_1^2}}{\rho _1}. \end{equation*}

Here ${\rho _1}$ is the fluid density in the background, and ${\rho _2}$ is that inside the cloak shell.

However, we still have two problems to tackle. Firstly, Equation (1) requires the fluid density inside the cloak to be compressed compared to that in the background. This contradicts the incompressibility of the ideal fluid as well as most real fluids in general. Secondly, Equation (1) is based on Laplace's equation, which applies only to the ideal fluid that has negligible viscosity. However, a real fluid must contain finite viscosity (an extremely low viscosity in a real fluid also comes along with an extremely high Reynold number (Re $\gg $ 1), marking the onset of turbulence). The influence of the viscosity remains an issue for the cloaking condition in Equation (1).

To tackle the problems mentioned above, we consider the creeping flow with a low Reynold number (Re $\ll $ 1) in a narrow gap between two plates, which is known as Hele-Shaw flow. In this case, a viscous flow can be simplified into an ideal fluid flow satisfying Laplace's equation [1]. Hence, the problem of viscosity can be circumvented. As illustrated in Fig. 1c, we consider fluid with a density ${\rho _1}$ that flows into a narrow channel with a height of ${h_{\rm{b}}}$. A solid cylinder with a radius ${R_1}$ that penetrates through the channel serves as a cylindrical obstacle. So, it can be expected that the viscous flow in the narrow channel in the presence of the cylindrical obstacle will behave like the picture in Fig. 1a (there will be some discrepancies in thin layers close to the boundary of the obstacle; to be discussed later).

Now we design the cloak. As mentioned above, it is impractical to compress the fluid density to fulfill the cloaking condition in Equation (1). However, we can emulate a higher local fluid density ${\rho _2}$ by extending the height ${h_{\rm{s}}}$ of the channel within the cloak shell region, as illustrated in Fig. 1c (see details in Supplementary Data). Changing the height of the fluid channel is practically feasible in many situations. For instance, the height of the Hele-Shaw cell has been engineered to control the precipitate patterns and viscous fingering [37,38]. In many microfluidic applications, the fluid channels are fabricated with 3D lithography [39], which can conveniently fabricate the cloak shell region with high resolution. According to mass conservation, the extended height required to construct a fluid-flow cloak satisfies the formula below:

(2) \begin{equation*} {h_{\rm{s}}} = \frac{{R_2^2 + R_1^2}}{{R_2^2 - R_1^2}}\ {h_{{\rm{b}}.}} \end{equation*}

It is worth mentioning that the value of ${h_{\rm{s}}}$ in Equation (2) is only an estimation. The no-slip condition (i.e. only zero velocity is allowed at the boundary) gives rise to distortion of streamlines in the Hele-Shaw flow in the vicinity of the boundaries of the obstacle, being different from the ideal fluid flow. Hence, the optimal height for the fluid-flow cloak should be slightly shifted from ${h_{\rm{s}}}$ calculated by Equation (2). The method we used to optimize ${h_{\rm{s}}}$ will be discussed in a later section.

**Figure 2.**
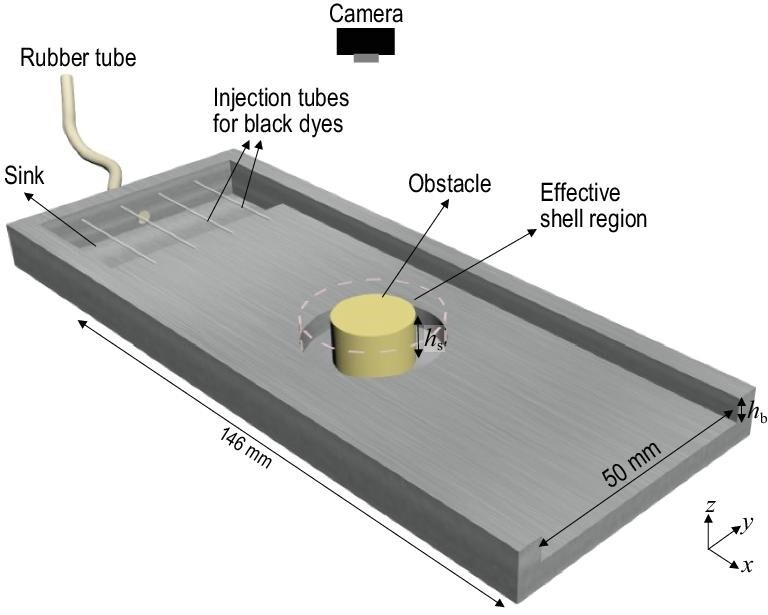
Schematic diagram of the experimental set-up. The glycerin that was dyed with white paint was first pumped into a sink through a wide rubber tube and then it flowed into a rectangular channel uniformly. The indicators, glycerin dyed with black dye, were injected into the channel manually with injection syringes through the injection tubes at a steady state. The flowing channel has a width of 50 mm and a length of 146 mm. A piece of glass plate was placed on top of the samples to enclose the channel. The shell region was extended downwards, like a trough surrounding the obstacle. The heights of the background and effective shell region are ${h_b}$ = 5 mm and ${h_s}$ = 10 mm, respectively. The entire process was recorded by a camera from the top.

Figure 2 shows the schematic diagram of the experimental set-up. We designed a rectangular channel with dimensions 146 mm $\times $ 50 mm $\times $ 5 mm. A cylindrical obstacle with a radius ${R_1}$ = 8 mm was placed at the center of the channel. As the fluid is affected by gravity, we extruded the cloak shell region with an outer radius ${R_2}$ = 14 mm along $- \hat{z}$ direction, like a trench surrounding the obstacle. The height of the shell region, measured from the top of the flowing channel to the bottom of the trench, is represented by ${h_{\rm{s}}}$. During the experiment, an electrically driven piston pump was used to pump the fluid into the set-up through a thick rubber tube. The fluid first filled up a sink and then flowed into the channel uniformly. Glycerin was used in our demonstration due to its high viscosity (∼$0.63\ {\rm{Pa}} \cdot {\rm{s}}$ for 95% glycerin solution at room temperature).

To achieve the scattering-cancellation-based fluid-flow cloak, the fluid flow must be governed by the Laplace equation, indicating that the Reynold number needs to be close to zero. In other words, high viscosity and low velocity are required to reach the limit of the Hele-Shaw flow approximation. The Reynold number is ∼0.08 in our case (see the calculations in Supplementary Data). Moreover, the diameter of the obstacle $2{R_1}$ = 16 mm is larger than the gap of the channel ${h_{\rm{b}}}$ = 5 mm, which is consistent with the Hele-Shaw flow [1].

Here we introduce our optimization method for the height of the cloak shell. The initial value of ${h_{\rm{s}}}$ = 9.85 mm is obtained from Equation (2). Therefore, the simulation, which uses the same set-up as the experiment, was repeated with a range of ${h_{\rm{s}}}$ close to 9.85 mm in COMSOL Multiphysics 5.2 in order to obtain the optimized ${h_{\rm{s}}}$. The creeping flow that is governed by Stokes equations was used in the simulation. The optimized ${h_{\rm{s}}}$ should show straight streamlines in the background region, as shown in Fig. 1b. Hence, we first extracted the *y*-coordinates of 40 streamlines, which were spaced equally in the *y*-direction, from each simulation. Next, we removed those *y*-coordinates that were far away from the obstacle or located inside the cloak shell region (see Supplementary Fig. 1). The standard deviation of remaining *y*-coordinates extracted from each streamline was calculated individually. Finally, we defined *y*-variation as the mean of these standard deviations of all streamlines.

**Figure 3.**
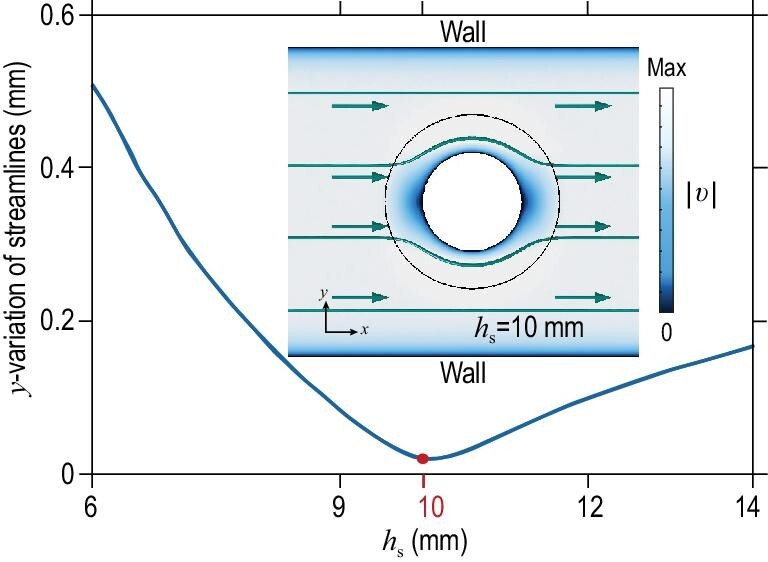
Optimization of the cloak geometry. The simulation, using the same set-up as the experiment, was repeated with different ${h_{\rm{s}}}$ in COMSOL Multiphysics 5.2. The minimum of the defined *y*-variation is obtained when ${h_{\rm{s}}}$ = 10 mm (represented by a red dot). The inset shows the simulation result when ${h_{\rm{s}}}$ = 10 mm. The color bar represents the magnitude of velocity. The circle with a black solid line shows the cloaking shell. The lines and arrows in a teal color represent the streamlines and the direction of the flow, respectively. The streamlines in the background region remain straight.

The dependence of *y*-variation on ${h_{\rm{s}}}$ is illustrated in Fig. 3, and the optimized ${h_{\rm{s}}}$, which should give the smallest *y*-variation, is shown to be 10 mm. Note that *y*-variation is very close to the minimum when ${h_{\rm{s}}}$ = 9.85 mm. The inset demonstrates the simulation result when ${h_{\rm{s}}}$ = 10 mm. The color bar represents the magnitude of total velocity. The lines and arrows in teal denote the streamlines and direction of flowing velocity, respectively. As anticipated, the streamlines are almost undisturbed in the background region.

## DEMONSTRATION OF THE FLUID-FLOW CLOAK

**Figure 4.**
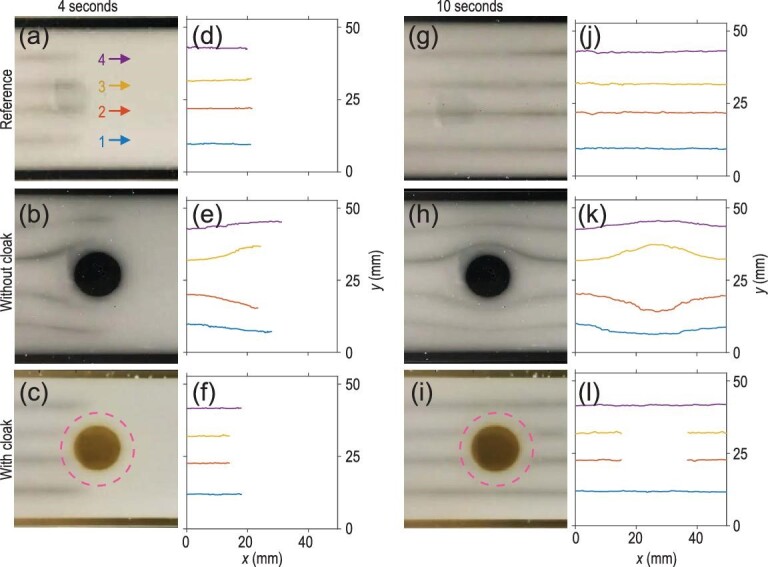
Experimental demonstration of the fluid cloak. (a–c) and (g–i) Observed streamlines at 4 seconds and 10 seconds, respectively. The fluid flowed from the left side of the photos. The streamlines are denoted by the indices from 1 to 4. (d–f) and (j–l) Streamlines extracted from (a–c) and (g–i) by an algorithm. The top row of (a), (d), (g) and (j) corresponds to the reference sample without the obstacle. The middle row of (b), (e), (h) and (k) corresponds to the obstacle sample without the cloak. The bottom row of (c), (f), (i) and (l) corresponds to the obstacle sample with the cloak. The cloak region is represented by a pink dotted circle in (c) and (i).

Three samples were prepared: (i) a reference sample without any obstacle, (ii) an obstacle sample without a cloak and (iii) an obstacle sample with a cloak. The movie provided in Supplementary Data recorded the dynamic process of the fluid flow passing by the obstacle. Despite the fact that the cloak is designed for steady states, we can see that the cloaking effect also works well for a dynamic scenario. We extracted snapshots at 4 seconds and 10 seconds of the video, as representative situations in the dynamic and steady cases (see illustrations in Fig. 4a–c and g–i). As mentioned before, the experiments were repeated with three samples and the glycerin was input from the left side of the snapshots. Four streamlines were visualized by the black indicators and labeled by indices ‘1’, ‘2’, ‘3’ and ‘4’ (Fig. 4a). For quantitative analysis, we also numerically traced the central position of each streamline, as plotted in Fig. 4d–f and j–l, which correspond to the snapshots in Fig. 4a–c and g–i, respectively.

Figure 4a, d, g and j verify that in the absence of any obstacle, the flow generated by the pump is uniform and the streamlines flow along straight trajectories with almost the same velocity. The vaguely visible shadows presented in Fig. 4a and g were just the reflections of the camera lens by the glass plate placed on top of the sample. By contrast, in the presence of the cylindrical obstacle, the fluid flow was blocked by the obstacle and the streamlines were deflected to the upper and lower sides of the obstacle (Fig. 4b, e, h and k). Although the side walls in our experiment might slightly reduce the distortion of streamlines in their vicinities, Fig. 4h and k still showed a similar pattern to Fig. 1a. Note that in Fig. 4b and e, the four streamlines were halfway in their detour around the obstacle. Because of the longer detour, the streamlines labeled ‘2’ and ‘3’ were slower than those labeled ‘1’ and ‘4’. This delay in the streamlines close to the obstacle is more evidence of the existence of the obstacle, apart from the apparent streamline deflection around the obstacle.

The results for the obstacle sample with the cloak, which demonstrate the realization of fluid cloaking, are shown in Fig. 4c, f, i and l. The cloaking shell region is denoted by a pink dotted circle. As anticipated, the distortion of streamlines outside the shell region was canceled and the streamlines were straight. The background region in Fig. 4i and l exhibited the same pattern as shown in Fig. 4g and j. Therefore, for the steady case, the cylindrical obstacle was effectively ‘hidden’ from the external fluid fluxes. For quantitative analysis, we extracted the coordinates of streamlines in Fig. 4j, k and l in the steady case and computed their *y*-variations. The *y*-variations, as shown in Fig. 4j (homogeneous fluid flow), k (bare obstacle without the cloak), and l (obstacle with the cloak), are 0.16 mm, 1.35 mm and 0.13 mm, respectively, which confirms the fluid-flow cloaking effect. Note that the *y*-variation is relatively large compared to the simulation as it is limited by the resolution in the measurement.

Interestingly, in Fig. 4c and f, as in the dynamic situation, when the streamlines labeled ‘2’ and ‘3’ reached the cloaking shell region, they stopped moving forward but would emerge after a while from another side of the cloak (as shown in Fig. 4i and l). That is because the cloak shell is a trench surrounding the obstacle. After entering the cloaking shell region, the dyes in the streamlines labeled ‘2’ and ‘3’, while making a detour in the *xy* plane around the obstacle, in fact lowered their heights in the *z*-direction; as a result, they disappeared in the video. After finishing the detour, the dyes in these streamlines returned to the initial heights when they left the cloaking shell region, and thus reappeared in the video.

**Figure 5.**
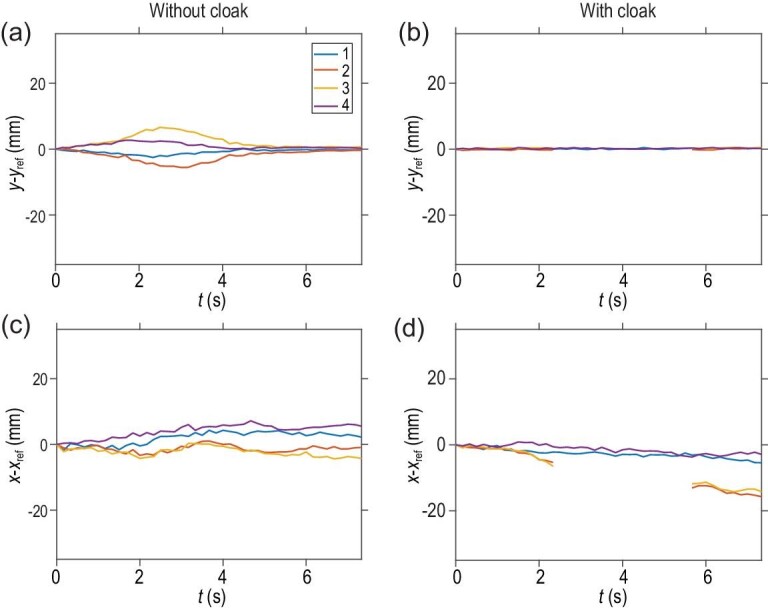
Time evolution analysis. (a) The difference in *y*-coordinates of streamline fronts between the obstacle sample without the cloak and the reference sample without the obstacle. (b) The difference in *y-*coordinates of streamline fronts between the obstacle sample with the cloak and the reference sample without the obstacle. (c) The difference in *x*-coordinates of streamline fronts between the obstacle sample without the cloak and the reference sample without the obstacle. (d) The difference in *x-*coordinates of streamline fronts between the obstacle sample with the cloak and the reference sample without the obstacle.

It is worth emphasizing that, although straight streamlines can be recovered by the cloak as if the obstacle did not exist, it does not mean the streamlines are completely unaffected. This can be demonstrated by showing the time evolution of the streamline fronts. Using the homogeneous fluid flow as the reference, we extracted the positions (i.e., x and y coordinates) of the streamline fronts at different moments and subtracted the measurement results with and without the cloak by those of the 4 reference points in Fig. 4a. Figure 5a and b show the time evolution of subtracted *y*-coordinates (*y* − *y*_ref_) in the absence and presence of the fluid-flow cloak, respectively. It can be seen from the *y-*coordinates in Fig. 5a that, in the absence of the cloak, the streamlines labeled ‘2’ and ‘3’ were significantly deflected downward and upward, respectively, while making the detour around the obstacle; in contrast, the streamlines labeled ‘1’ and ‘4’ were deflected less from the obstacle as compared with the streamlines `2' and `3'. In the presence of the cloak, on the other hand, the subtracted *y-*coordinates of streamline fronts were all flat, as shown in Fig. 5b, due to the recovered straight streamlines. The only difference is the missing parts of streamlines ‘2’ and ‘3’ from ∼2.5 seconds to 5.5 seconds, as they entered and left the cloaking shell region. Therefore, by observing only the time evolution of *y*-coordinates, it is difficult to tell the existence of the obstacle that was cloaked.

However, the existence of the obstacle can still be revealed by monitoring the time evolution of *x*-coordinates. As shown in Fig. 5c, in the absence of the cloak, the detour of streamlines in the cloaking shell region caused a time delay for the streamlines labeled ‘2’ and ‘3’, as compared to streamlines ‘1’ and ‘4’. This time delay remained even after all streamlines had left the cloaking shell region. Note that because of the fluid flowing along the *x*-direction, the measured curves of *x*-coordinates were less smooth compared to those of *y*-coordinates, but the relative positions, namely the time delay, among *x*-coordinates, were still apparent.

Now we check the time evolution of *x*-coordinates for the obstacle sample with the cloak. Figure 5d shows that the time delay of streamlines ‘2’ and ‘3’ compared to streamlines ‘1’ and ‘4’ was preserved, even after all streamlines had left the cloaking shell region. Therefore, by checking the time delay of streamlines, it was still possible to detect the existence of the obstacle. This kind of time delay is an intrinsic limitation to all practical cloaking devices, such as those for electromagnetic waves [40]. The time delay can only be canceled by increasing the fluid-flow velocity in the cloak region using certain propulsion systems (e.g. mini pumps). Despite this time delay limitation, the fluid-flow cloak was still able to effectively hide the obstacle by recovering the straight streamlines.

In contrast to the optical cloak, there is no overall size limit to the fluid-flow cloak as the fluid flow is always governed by the Laplace equation, which is independent of the size. In other words, the proposed approach can be applied to cloaking an object of arbitrary size and shape in a fluid channel. The only prerequisite of our proposal is that the height of the fluid channel is smaller than the radius of the obstacle as well as the width of the fluid chamber, in order to achieve the Hele-Shaw flow. This requirement makes our approach useful for microchannels where the height of the fluid channel is intrinsically small.

## CONCLUSION

In summary, our work demonstrates an innovative approach to realizing a fluid-flow cloak that can hide a cylindrical obstacle in a narrow fluid channel. In contrast to previous designs employing complex metamaterials, our fluid cloak adopts the scattering cancellation approach and is realized by merely adjusting the geometry of the fluid channel. Such a simple solution is applicable to any fluid flow that can be approximated as an ideal fluid flow. The proposed fluid-flow cloak can serve as a new strategy in fluid-flow control, especially in the application of microfluidic channels, where a Hele-Shaw flow takes effect. We envision that such fluid-flow cloaks can be used to minimize the distortion caused by devices (detectors, heaters) that have to be in contact with the fluid.

## METHODS

The samples were manufactured through 3D printing. A piece of glass plate was placed on top of the samples to enclose the flow channel. The indicators, the glycerin dyed with black acrylic paint, were injected manually through four evenly distributed thin rubber tubes to visualize the streamlines. Apart from that, the background fluid was dyed with white acrylic paint to improve the visualization of the streamlines. The experiments were recorded by a camera from the top. The simulations were conducted in COMSOL Multiphysics.

## Supplementary Material

nwab205_Supplemental_Files
