# Supplementary material for: A metamaterial-free fluid-flow cloak
Source: Natl Sci Rev. 2021 Nov 17;9(9):nwab205. doi: 10.1093/nsr/nwab205 (PMC9555300; doi:10.1093/nsr/nwab205)
Supplement: nwab205_Supplemental_Files [file nwab205_supplemental_files.zip › 211107_NSR_SM_A_metamaterial-free_fluid_flow_cloak_manuscript.docx]

**Supplementary data**

A Metamaterial-Free Fluid Flow Cloak

Fuyang Tay^1,#^, Youming Zhang^1,#^, Hongyi Xu^1,#^, Honghui Goh^1^, Yu Luo^2,(a)^ and Baile Zhang^1,3,(b)^

**1. Derivation of the fluid cloak**

The ideal fluid flow is governed by the continuity equation, $\frac{\partial\rho}{\partial t}+\nabla\cdot\left( \rho v \right)=0.$ As mentioned in the article, the velocity of ideal fluid flow can be described by a velocity potential such that $v=\nabla\phi$. In addition, $\rho$ is constant if we assume the fluid is incompressible. Therefore, at a steady state where $\frac{\partial\rho}{\partial t}=0$, the final expression is simply a Laplace’s equation,

$$\begin{aligned} \rho\nabla^{2}\phi=0. \#\left( S1 \right) \end{aligned}$$

Equation (S1) is in the same form as the corresponding physical field in previous magnetic/thermal cloaks.

Here we consider the invisibility cloak design in Fig. 1b. We use $(r,\theta,z)$ to represent cylindrical coordinates and index $i$ to represent different regions: (i) $i=0$ as the region inside the obstacle ($r<R_{1}$); (ii) $i=1$ as the region in the background region ($r>R_{2})$; (iii) $i=2$ as the cloaking shell region $(R_{1}<r<R_{2}$). Note that $\rho_{0}=0$ as no fluid inside the obstacle and $\rho_{1}$ and $\rho_{2}$ are the fluid density in the background region and cloaking shell region respectively. We assume there is no variation in $z$-direction, and the velocity potential in different regions can be expressed in the form

$$\begin{aligned} \phi_{0}=Ar\cos\theta, \#\left( S2 \right) \end{aligned}$$

$$\begin{aligned} \phi_{2}=\left( Br+\frac{C}{r} \right)\cos\theta, \#\left( S3 \right) \end{aligned}$$

$$\begin{aligned} \phi_{1}=\left( Dr+\frac{E}{r} \right)\cos\theta. \#\left( S4 \right) \end{aligned}$$

The relations between the coefficients ($A$-$E$) can be obtained by solving the boundary conditions,

$$\begin{aligned} AR_{1}^{2}-BR_{1}^{2}-C=0, \#\left( S5 \right) \end{aligned}$$

$$\begin{aligned} BR_{1}^{2}-C=0, \#\left( S6 \right) \end{aligned}$$

$$\begin{aligned} BR_{2}^{2}+C-DR_{2}^{2}-E=0, \#\left( S7 \right) \end{aligned}$$

$$\begin{aligned} \rho_{2}BR_{2}^{2}-\rho_{2}C-\rho_{1}DR_{2}^{2}+\rho_{1}E=0, \#\left( S8 \right) \end{aligned}$$

To achieve the cloaking effect, we set $E=0$ such that the flowing velocity outside the cloaking shell $v=\nabla\phi_{1}=D\hat{x}$ where $D$ is a constant, in the presence of the cylindrical obstacle. Finally, the relation between $\rho_{1}$ and $\rho_{2}$ is given as

$$\begin{aligned} \rho_{2}=\frac{R_{2}^{2}+R_{1}^{2}}{R_{2}^{2}-R_{1}^{2}}\rho_{1}. \#\left( S9 \right) \end{aligned}$$

The cloak design with higher fluid density is shown in Supplementary Fig. 1(a), whose 2D plot is shown in Fig. 1(a) in the main text. However, it is not practical to increase the fluid density locally. Therefore, the design in Supplementary Fig. 1(b) is adopted to realize the fluid flow cloak. We would like to highlight that, in Supplementary Fig. 1(b), the actual fluid density remains as $\rho_{1}$ in the channel, irrespective of the channel height. The total mass inside the cloaking region is given by $m_{\mathrm{cloak}}=\rho_{1}\times\pi\left( R_{2}^{2}-R_{1}^{2} \right)h_{s}$. Nevertheless, from the perspective of the effective medium, the height of the cloak region is still interpreted as $h_{b}$. For instance, Supplementary Fig. 1(c) illustrates the cross-section of fluid flowing through a channel with a decreasing height. The effective fluid density, $\rho_{\mathrm{eff}}$, in *xy*-plane at different *x* coordinates is proportional to the fluid mass inside a red rectangle with a fixed height. Therefore, $\rho_{\mathrm{eff}}$ is dependent on the channel height, and $\rho_{eff,1}>\rho_{eff,2}>\rho_{eff,3}$ in Supplementary Fig. 1(c). Since the total mass has to be conserved, the effective mass density of the cloak region is then obtained as $\rho_{2}=\frac{m_{\mathrm{cloak}}}{\pi\left( R_{2}^{2}-R_{1}^{2} \right)h_{b}}=\frac{\rho_{1}h_{s}}{h_{b}}$. In other words, increasing $h_{s}$ gives rise to larger effective mass density $\rho_{2}$. Therefore, equation (S9) can be rewritten as

$$\begin{aligned} h_{s}=\frac{R_{2}^{2}+R_{1}^{2}}{R_{2}^{2}-R_{1}^{2}}h_{b}.\#(S10) \end{aligned}$$

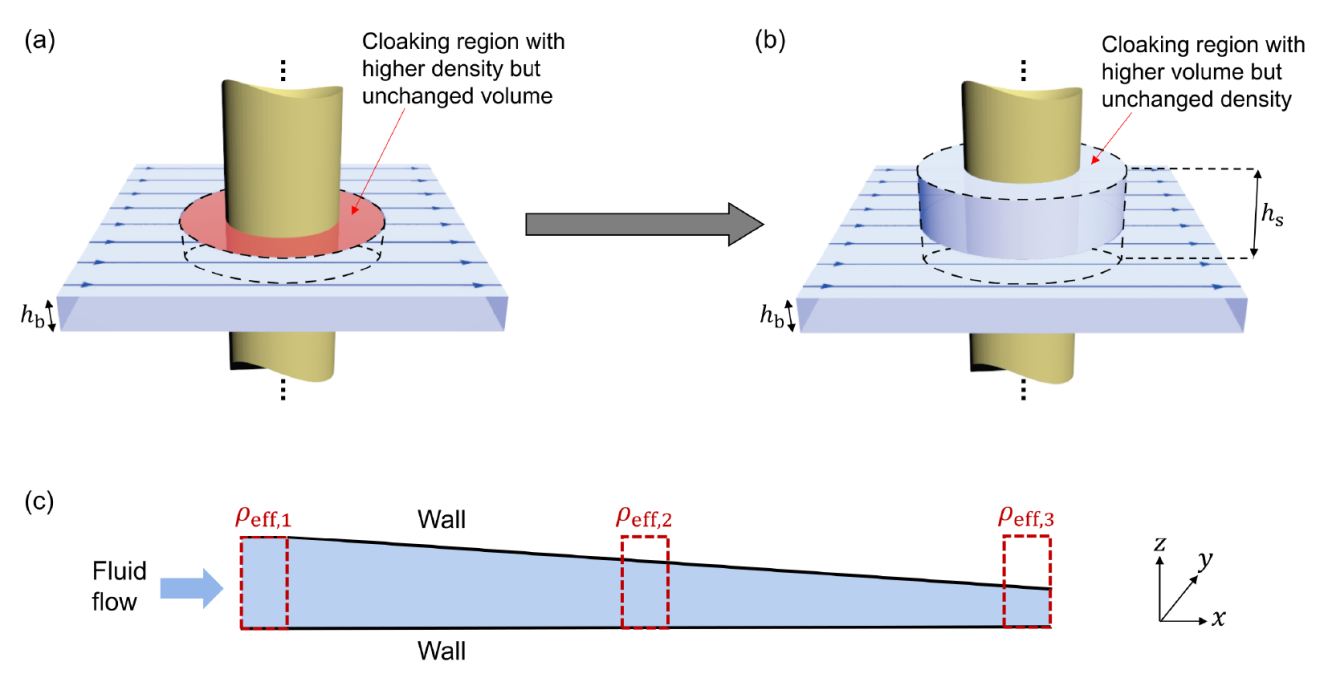


**Supplementary Figure 1.** The step of emulating a higher fluid density with a higher volume. (a) The hypothetical design that requires a higher local fluid density, given that the volume is unchanged. (b) The practical design that has a higher volume (equivalent to a higher height), given that the fluid density is unchanged. (c) Schematic diagram of fluid flowing along the *x* direction through a channel with a decreasing height. The effective fluid density in *xy*-plane, $\rho_{\mathrm{eff}}$, is dependent on the channel height. $\rho_{eff,1}>\rho_{eff,2}>\rho_{eff,3}$.

**2. Calculation of Reynolds number**

To verify the flow pattern in our experiment, we calculated the Reynolds number, $Re=\frac{\rho vD}{\mu}$, where $\rho$ is the density of the glycerin (= 1261$kg/m^{3}$), $v$ is the flowing velocity (~ 0.008 m/s), $D$ is the height of the rectangular channel ($h_{b}$ = 5 mm) and $\mu$ is the dynamic viscosity (= 0.63 Pa$\cdot$s). Thus, $Re\approx$ 0.08$\ll$1, which satisfies the condition of creeping flow.

**3. Optimization method of** $\boldsymbol{h}_{\mathbf{s}}$

Here we introduce the method to get the optimized $h_{s}$ for the fluid cloak. We first plotted 40 streamlines that were spaced equally along $y$-direction at $x$ = -50 mm and $z$ = 0 mm, as shown in Supplementary Fig. 2 (blue lines). We extracted the $y$-coordinates of each streamline from the COMSOL. Next, we removed those $y$-coordinates which were (i) far away from the obstacle, for example, $\left| x \right|$ > 50 mm and (ii) located inside the cloaking shell region such as $\sqrt{x^{2}+y^{2}}$ < 14 mm. So, only $y$-coordinates of streamlines within the red shaded region were extracted. Finally, we calculated the standard deviations of remaining $y$-coordinates of each streamline separately and we defined $y$-variation as the mean of these standard deviations. As expected, streamlines within the red shaded region in the obstacle sample with a fluid cloak exhibit the same pattern as those in the reference sample.

The minimum of the $y$-variation of the streamlines, which remains a finite value, arises mainly from the distortion caused by the “no-slip” (i.e., zero velocity) boundary condition imposed at the walls of the channel and the boundaries of the cloak. As seen in the inset of Fig. 3 in the main text, the flowing velocity is lower (represented by darker colors) near to the wall and the obstacle due to the boundary effect. This boundary effect is a natural result of the finite viscosity, which is not considered in the ideal fluid model, and thus cannot be cancelled with the scattering cancellation approach.


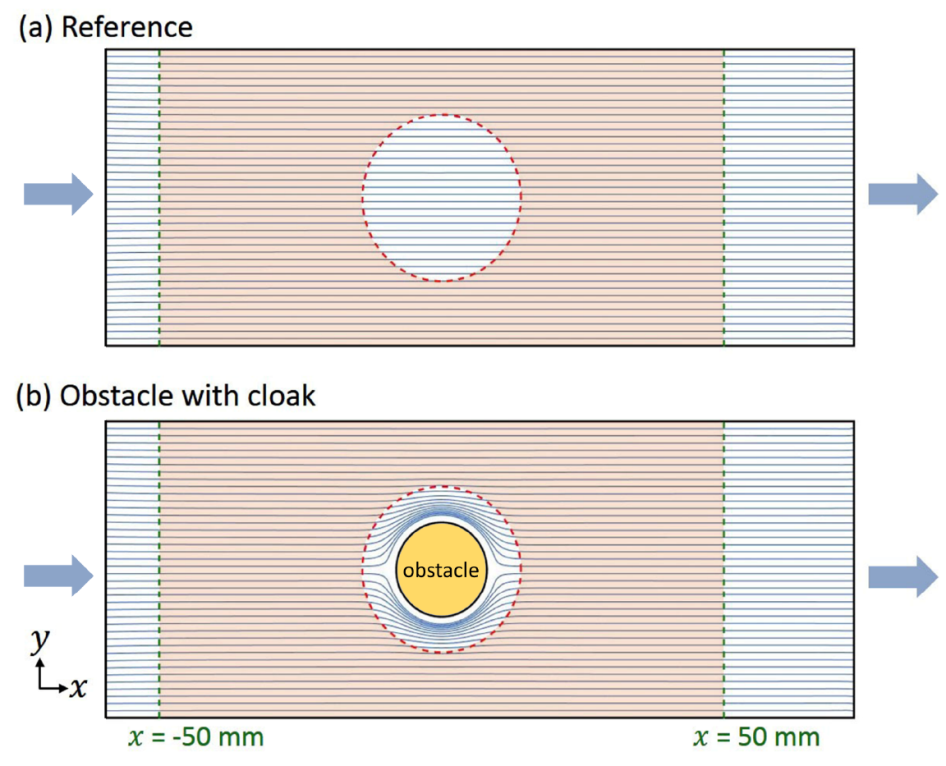


**Supplementary Figure 2.** Extracted streamlines in simulation. The fluid flows through (a) a reference sample and (b) an obstacle sample with the cloak while $h_{s}$ = 10 mm. The black solid lines and red dashed circle represent the walls and the cloaking shell region with outer radius, $R_{2}$ = 14 mm respectively. Only $y$-coordinates of streamlines within the red shaded region are extracted and the mean standard deviations of $y$-coordinates of each streamline should be the smallest for optimized $h_{s}$.

**4. Extraction of streamlines coordinates**

We converted the snapshots into grayscale and defined the confidence intervals of color value for different regions. Only the *x* and *y* coordinates with color values within the confidence interval were then extracted. Finally, we averaged the y coordinates for each x coordinate to obtain the trajectory of each streamline.

**5. Streamlines as a function of** $\boldsymbol{z}$

The simulation results confirm that the streamlines are independent of the channel depth, $z$. The streamlines outside the cloak region starting at different heights are uniform in the presence of the cloak.


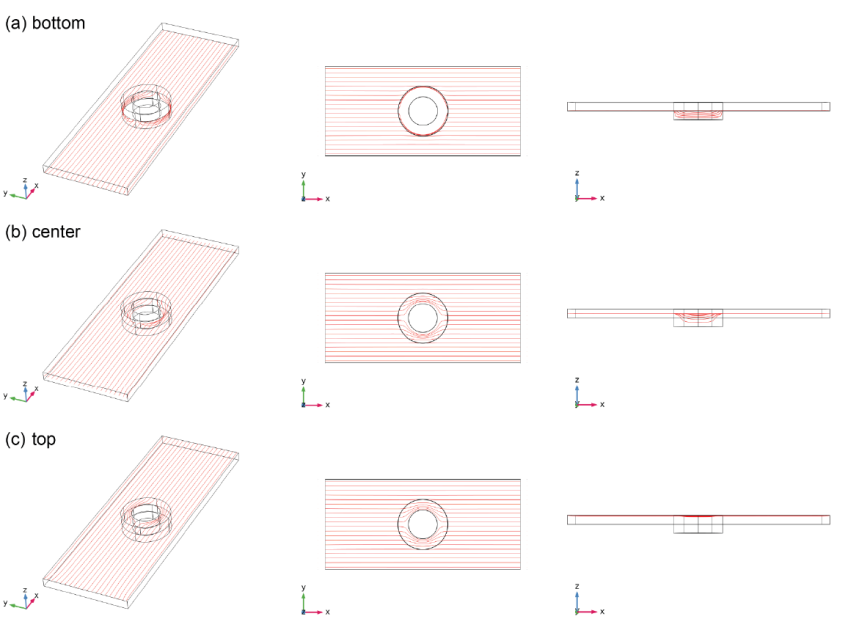


**Supplementary Figure 3.** Streamlines starting at (a) the bottom of the channel, (b) the center of the channel and (c) the top of the channel. Different columns represent the streamlines from different views. The fluids are flowing along the *x*-direction. The streamlines outside the cloak region are uniform at all heights.

**6. Simulations when the obstacle is filled with fluid**

In our fluid-flow cloak, the inner boundary needs to be solid and impenetrable. This solid and impenetrable inner boundary is able to isolate the cloaked region from the external environment. In other words, any object that is placed inside the cloaked region will not affect the straight streamlines in the external fluid flow. Supplementary Fig. 4 shows two situations when the cloak is filled with fluid: (a) the inner boundary of the cloak still exists; and (b) the inner boundary of the cloak is removed. In situation (a), the external streamlines will not be distorted. However, in the situation (b), they will be distorted because of the absence of the inner boundary.


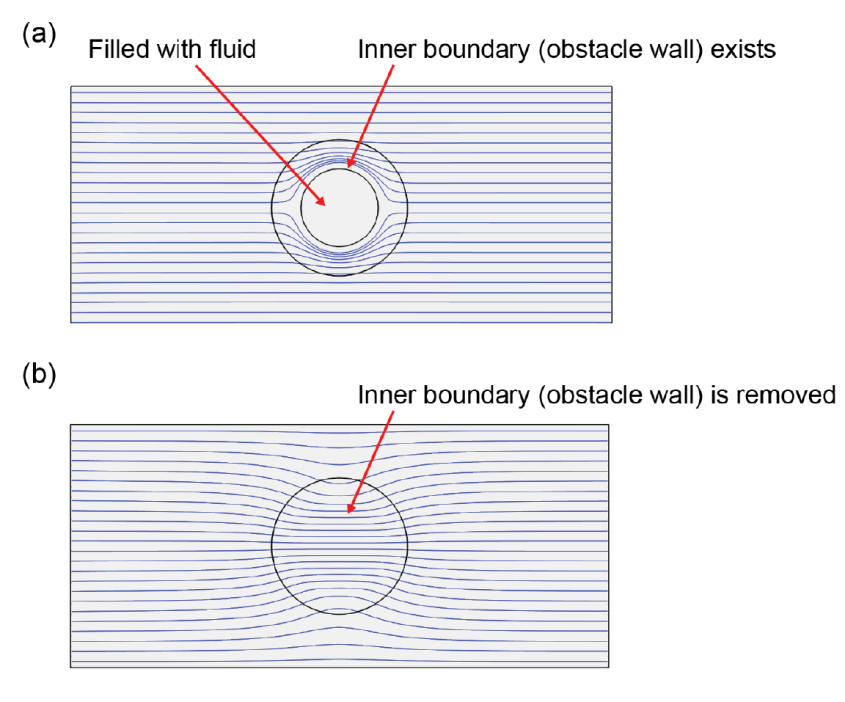


**Supplementary Figure 4.** Simulation results when the obstacle is filled with the fluid if (a) the inner boundary of the cloak exists and (b) the inner boundary of the cloak is removed. Blue lines represent the streamlines. The streamlines outside the cloaking regions will not be distorted only if the inner boundary of the cloak exists.
